# Effects of terlipressin as early treatment for protection of brain in a model of haemorrhagic shock

**DOI:** 10.1186/s13054-015-0825-9

**Published:** 2015-03-13

**Authors:** Keila Kazue Ida, Denise Aya Otsuki, Adolfo Toshiro Cotarelli Sasaki, Emilyn Silva Borges, Letícia Urbano Cardoso Castro, Talita Rojas Sanches, Maria-Heloisa Massola Shimizu, Lúcia Conceição Andrade, José-Otávio Costa Auler, Alex Dyson, Kenneth John Smith, Joel Avancini Rocha Filho, Luiz-Marcelo Sá Malbouisson

**Affiliations:** Laboratório de Investigação Médica (LIM-08), Disciplina de Anestesiologia, Faculdade de Medicina, Universidade de São Paulo, Avenida Doutor Arnaldo, 455, 2° andar, sala 2120, Cerqueira César, São Paulo, SP 01246-903 Brazil; Department of Neuroinflammation, Institute of Neurology, University College London (UCL), 1 Wakefield Street, 2nd floor, WC1N 1PJ, London, UK; Disciplina de Nefrologia, Faculdade de Medicina, Universidade de São Paulo (LIM-12 HC-FMUSP), Avenida Doutor Arnaldo, 455, 3rd floor, Cerqueira César, São Paulo, SP 01246-903 Brazil; Division of Medicine, University College London (UCL), Gower Street, WC1E 6BT, London, UK; Divisão de Anestesiologia, Hospital das Clínicas da Faculdade de Medicina da Universidade de São Paulo (HCFMUSP), Av. Dr. Enéas de Carvalho Aguiar,155, 8th floor, 05403-000, São Paulo, SP Brazil

## Abstract

**Introduction:**

We investigated whether treatment with terlipressin during recovery from hypotension due to haemorrhagic shock (HS) is effective in restoring cerebral perfusion pressure (CPP) and brain tissue markers of water balance, oxidative stress and apoptosis.

**Methods:**

In this randomised controlled study, animals undergoing HS (target mean arterial pressure (MAP) 40 mmHg for 30 minutes) were randomised to receive lactated Ringer’s solution (LR group; *n* =14; volume equal to three times the volume bled), terlipressin (TERLI group; *n* =14; 2-mg bolus), no treatment (HAEMO group; *n* =12) or sham (*n* =6). CPP, systemic haemodynamics (thermodilution technique) and blood gas analyses were registered at baseline, shock and 5, 30, 60 (T60), 90 and 120 minutes after treatment (T120). After the animals were killed, brain tissue samples were obtained to measure markers of water balance (aquaporin-4 (AQP4)), Na^+^-K^+^-2Cl^−^ co-transporter (NKCC1)), oxidative stress (thiobarbituric acid reactive substances (TBARS) and manganese superoxide dismutase (MnSOD)) and apoptotic damage (Bcl-x and Bax).

**Results:**

Despite the HS-induced decrease in cardiac output (CO) and hyperlactataemia, resuscitation with terlipressin recovered MAP and resulted in restoration of CPP and in cerebral protection expressed by normalisation of AQP4, NKCC1, TBARS and MnSOD expression and Bcl-x/Bax ratio at T60 and T120 compared with sham animals. In the LR group, CO and blood lactate levels were recovered, but the CPP and MAP were significantly decreased and TBARS levels and AQP4, NKCC1 and MnSOD expression and Bcl-x/Bax ratio were significantly increased at T60 and T120 compared with the sham group.

**Conclusions:**

During recovery from HS-induced hypotension, terlipressin was effective in normalising CPP and cerebral markers of water balance, oxidative damage and apoptosis. The role of this pressor agent on brain perfusion in HS requires further investigation.

## Introduction

Haemorrhagic shock is the leading cause of early death in trauma patients [1]. During the pre-hospital period, haemorrhage contributes to death in 33% to 56% of cases, and it is the most common cause of death among those found dead upon the arrival of emergency medical services personnel [2]. Neurological signs such as altered mental state, which typically includes obtundation, disorientation, confusion, agitation and irritability, cannot be neglected, because cerebral hypoperfusion is a consequence in patients experiencing bleeding-associated hypotension [3-5]. In addition, animal studies of haemorrhagic shock have shown that cerebral ischaemia with cell damage begins at the onset of the haemodynamic impairment [6-8].

Under hypotensive conditions such as those in haemorrhagic shock, cerebral perfusion pressure (CPP) is sustained below the lower limits of autoregulation [8], which is detrimental to brain tissue oxygenation [5,7,9]. Cerebral ischaemia has been associated with dysregulation of aquaporin-4 (AQP4) and Na^+^-K^+^-2Cl − co-transporter (NKCC1) in the astrocytes [9,10] and Bcl-2 related apoptotic proteins in the neurons [11]. Oxidative stress is implicated in the neuronal apoptosis that occurs in haemorrhagic shock. It has been shown to accompany increased lipid peroxidation within the brain, as reflected by changes in the levels of thiobarbituric acid reactive substances (TBARS) and changes in the expression of antioxidant enzymes such as manganese superoxide dismutase (MnSOD) [12].

Standard resuscitation practice for haemorrhagic shock mandates use of high-volume crystalloids. However, such therapy can result in adverse effects such as interstitial oedema in the gut and cellular oedema in the heart [13], increases in the inflammatory cytokine profile [14] and increased intracranial pressure (ICP) [8]. Crystalloids may also fail to recover CPP and oxygenation within the brain [8,15]. Terlipressin is a synthetic, long-acting (4 to 6 hours) analogue of vasopressin. The structure of terlipressin contains a peptide that represents the natural hormone lysine vasopressin, the innate vasopressin analogue in pigs. Its structure is very similar to human arginine vasopressin, but the synthetic drug is characterised by a more specific V_1_ agonistic effect (V_1_:V_2_ ratio =2.2:1) compared with arginine-vasopressin (V_1_:V_2_ ratio =1:1). Terlipressin has been studied as a vasoactive drug in the management of catecholamine-resistant arterial hypotension in septic shock [16], liver failure [17] and acute gastrointestinal bleeding [18]. The effects of terlipressin consist of vasoconstrictive activity on vascular smooth muscle cells and a pronounced vasoconstriction within the splanchnic circulation that has been shown to redistribute blood flow to recover perfusion pressure to organs such as the liver, kidney and brain [19,20] and to increase survival rates in animal studies of haemorrhagic shock [14,21]. It has been reported that terlipressin can improve CPP in patients with acute liver failure [17], septic shock [22] and traumatic brain injury with catecholamine-resistant shock [23]. However, the effects of terlipressin on cerebral haemodynamics during early treatment for haemorrhagic shock remain unclear, and no data are available comparing the effects of resuscitation with terlipressin with those of standard fluid.

We hypothesised that early recovery of haemorrhagic shock-induced hypotension with terlipressin could restore CPP and improve oxygenation of the brain. Therefore, the purpose of the present study was to investigate the effects of early administration of terlipressin on CPP and brain tissue oxygen pressure (PbtO_2_), as well as on the regulation of tissue markers of water balance (that is, AQP4 and NKCC1), oxidative stress (that is, TBARS and MnSOD) and apoptosis (that is, Bax and Bcl-x) within the brain in a porcine model of haemorrhagic shock.

## Materials and methods

### Ethical approval

This prospective randomised experimental study was approved by the Comissão de Ética em Pesquisa of the Hospital das Clínicas at Faculdade de Medicina of Universidade de São Paulo (067/11 and 280/13).

### Surgical preparation and monitoring

Female Large White pigs (*n* =46) weighing 20 to 30 kg were fasted for 12 hours with free access to water before the experiments. The animals were premedicated with ketamine (5 mg/kg intramuscular) and midazolam (0.25 mg/kg intramuscular), and anaesthesia was induced with propofol (7 mg/kg intravenous). After endotracheal intubation, anaesthesia was maintained with isoflurane vaporized in 40% oxygen, and the pigs were ventilated (Fabius GS Premium; Dräger, Lübeck, Germany) with a tidal volume of 8 ml/kg and positive end-expiratory pressure of 5 cmH_2_O. The respiratory rate was adjusted to maintain normocapnia (partial pressure of carbon dioxide in arterial blood (PaCO_2_) between 35 and 45 mmHg). Lactated Ringer’s solution (4 ml/kg/hr) and pancuronium (0.3 mg/kg/hr) were administered continuously throughout the experiments. Body temperature was maintained at 38°C using a heated mat (Medi-Therm II; Gaymar Industries, Orchard Park, NY, USA).

Both femoral arteries were catheterised for measurement of mean arterial pressure (MAP) and withdrawal of blood to induce haemorrhagic shock, respectively. Another catheter was inserted into the right femoral vein for later administration of treatments.

A 7.5-French pulmonary artery catheter (Swan-Ganz; Edwards Lifesciences, Irvine, CA, USA) was surgically introduced into the right internal jugular vein and advanced under continuous pressure recording into wedge position. Cardiac output was determined by bolus pulmonary artery thermodilution (Vigilance monitor; Edwards Lifesciences). All catheters and pressure transducers were filled with isotonic saline containing heparin (5 U/ml) and connected to a multiparametric data collection system (IntelliVue MP50 monitor; Philips Healthcare, Best, the Netherlands). The heart rate (HR), right atrial pressure (RAP), mean pulmonary artery pressure (MPAP), pulmonary artery occlusion pressure (PAOP) and central body temperature were also continuously monitored with the IntelliVue MP50 monitor.

The cardiac index (CI) was calculated to normalise the data for body surface area in square metres by using a conversion factor appropriate for pigs (*k* × BW^2/3^, where *k* =0.09) [24]. Systemic vascular resistance index (SVRI), pulmonary vascular resistance index (PVRI), left ventricular stroke work index (LVSWI), right ventricular stroke work index (RVSWI), stroke volume index (SVI), systemic oxygen delivery index (DO_2_I), systemic oxygen consumption (VO_2_I) and systemic oxygen extraction ratio (O_2_ER) were calculated using standard formulae [25].

Arterial and mixed venous blood were sampled at each time point for blood gas analysis, including measurement of haemoglobin (Hb), lactate, sodium (Na^+^) and potassium (K^+^) ion levels (ABL 555 blood gas meter; Radiometer, Copenhagen, Denmark).

Two burr holes of 5-mm diameter each were placed over the right and left coronal sutures (12-mm paramedian). In the right hemisphere, an intraparenchymal probe was inserted into the cerebral cortex (15-mm depth) and secured with a single lumen bolt for measurement of PbtO_2_ (Neurovent-PTO; RAUMEDIC, Helmbrechts, Germany). On the left side, a fibre-optic probe (Codman ICP EXPRESS Monitoring System; Codman Neuro, Raynham, MA, USA) was inserted epidurally for continuous monitoring of ICP after sealing the cranial window with bone wax. CPP was calculated using a standard formula (CPP = MAP − ICP) [6].

### Experimental design

Following surgical preparation, animals were allowed to stabilise for 30 minutes before being randomly divided into one of the following four groups: (1) a sham group (*n* =6) consisting of animals that were not subjected to haemorrhagic shock, (2) a HAEMO group (*n* =12) that was subjected to haemorrhagic shock and did not receive treatment, (3) a LR group (*n* =14) that was subjected to haemorrhagic shock and treated with LR (volume equal to three times the volume bled) and (4) a TERLI group (*n* =14) that was subjected to haemorrhagic shock and treated with terlipressin (2-mg bolus of GLYPRESSIN; Ferring Pharmaceuticals, São Paulo, Brazil).

Randomisation was previously performed, and the blind allocation of the pigs among groups was placed in numbered manila envelopes, which were opened in a consecutive manner immediately before baseline measurements were registered.

Haemorrhagic shock was induced by pressure-controlled bleeding targeting a MAP of 40 mmHg, which was maintained for 30 minutes before treatment. Data were recorded prior to blood removal (baseline), at 30 minutes after achieving the target MAP (shock) and at 5 minutes (T5), 30 minutes (T30) and 60 minutes (T60) after treatment. In some of these animals (sham group: *n* =3; HAEMO group: *n* =9; LR group: *n* =9; TERLI group: *n* =9), the study was continued for 1 additional hour, allowing data to be registered at 90 minutes (T90) and 120 minutes (T120) posttreatment. At the end of the study, the animals were killed with an overdose of isoflurane and potassium chloride. The intraparenchymal probe then was macroscopically inspected for insertion depth, and cortical samples of the brain were collected and immediately frozen in liquid nitrogen and stored at −80°C for later analysis.

### Preparation of cerebral samples for Western blotting assays and thiobarbituric acid reactive substance measurement

The samples were homogenized in ice-cold solution (200 mM mannitol, 80 mM 4-(2-hydroxyethyl)piperazine-1-ethanesulfonic acid, 41 mM KOH, pH 7.5) containing a protease inhibitor cocktail (Sigma-Aldrich, St Louis, MO, USA) using a POLYTRON PT 10–35 homogenizer (KINEMATICA, Lucerne, Switzerland). The homogenates were centrifuged at 4,000 × *g* for 30 minutes at 4°C to remove cell debris. Protein concentrations were determined by the Bradford assay method using a Bio-Rad protein assay kit (Bio-Rad Laboratories, Hercules, CA, USA).

### Western blotting

Western blotting assays were performed to assess the expression of the following proteins: AQP4, NKCC1, Bcl-x, Bax and MnSOD. Cerebral tissue samples were run on 12% polyacrylamide minigels for AQP4, Bax, Bcl-x and MnSOD and on 8% polyacrylamide minigels for NKCC1. After transfer by electroelution to polyvinylidene fluoride membranes (Amersham Hybond-P; GE Healthcare Life Sciences, Little Chalfont, UK), blots were blocked with 5% non-fat milk and 0.1% Tween 20 in Tris-buffered saline for 1 hour. Blots were then incubated overnight with an anti-AQP4 antibody (1:2,000), NKCC1 (1:500), Bcl-x antibody (1:500), Bax antibody (1:500) and MnSOD antibody (1:200). The labelling was visualised with a horseradish peroxidase-conjugated secondary antibody (anti-rabbit immunoglobulin G (IgG), diluted 1:2,000; anti-goat IgG, diluted 1:10,000; anti-mouse IgG diluted 1:2,000; or anti-mouse IgG, diluted 1:2,000, respectively; Sigma-Aldrich) using an enhanced chemiluminescence (ECL) detection system (Amersham ECL Western Blotting Detection kit; GE Healthcare Life Sciences). The ECL membranes were scanned using Alliance 4.2 (UVItec, Cambridge, UK), and the cerebral AQP4, NKCC1, Bcl-x and Bax protein expression levels were quantified using densitometry, normalizing the bands to actin expression.

### Thiobarbituric acid assay

To assess the levels of TBARS, a 0.2-ml cortical cerebral homogenate sample was diluted in 0.8 ml of distilled water, followed by addition of 1 ml of 17.5% trichloroacetic acid. Then, 1 ml of 0.6% thiobarbituric acid, pH 2, was added to the sample and placed in a boiling-water bath for 15 minutes. After the sample was allowed to cool, 1 ml of 70% trichloroacetic acid was added and the mixture was incubated for 20 minutes. The sample was then centrifuged at 2,000 × *g* for 15 minutes. The absorbance was recorded at 534 nm using a spectrophotometer, and values were calculated by using a molar extinction coefficient of 1.56 × 105 M/cm. The TBARS levels were then normalised to the total protein concentration, and the results are expressed as nanomoles per gram of protein.

### Statistical analysis

Physiological and neuromonitoring parameters and arterial and mixed venous blood gas data were analysed (GraphPad Prism version 5.03 for Windows; GraphPad Software, La Jolla, CA, USA) across groups and time using two-way analysis of variance (ANOVA) tests. Tukey’s tests were used for *post hoc* analysis. Analyses of survival were performed according to the Kaplan–Meier method and compared using Fisher’s exact test. The last observation carried forward imputation method was applied throughout the study for the animals that died. Differences in the expression levels of AQP4, NKCC1, Bcl-x, Bax, MnSOD and TBARS were analysed by one-way ANOVA followed by the Student-Newman-Keuls test, and the results are presented as mean ± standard error. For all analyses, *P <*0.05 was considered statistically significant.

## Results

**Figure 1:**
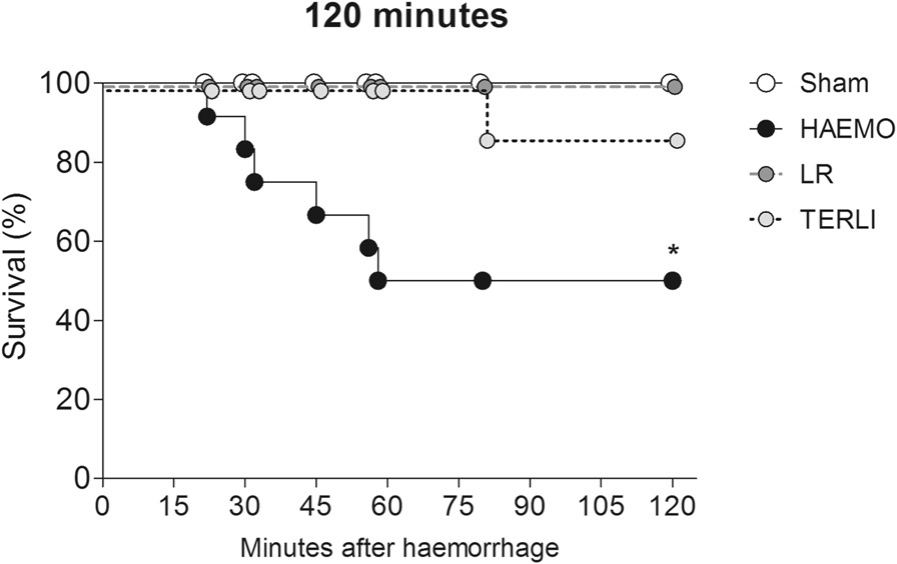
**Percent survival at 120 minutes after haemorrhagic shock using the Kaplan–Meier curve.** Rats treated with lactated Ringer’s solution (LR) and terlipressin (TERLI) after haemorrhagic shock were compared with the non-treated rats (HAEMO) and a sham group that was not subjected to bleeding. *P =0.0007 indicates significant difference compared with the sham group.

The blood volume withdrawn from each group was similar, averaging 60% of the estimated blood volume (HAEMO group: 1,083 ± 124 ml; LR group: 1,162 ± 203 ml; TERLI group: 1,011 ± 215 ml). In the HAEMO group, the number of deaths following haemorrhage was significantly higher at 120 minutes (six deaths at 41 ± 15 minutes after shock; *P* =0.0007). At 120 minutes after shock, one animal from the TERLI group had died (at 80 minutes after shock) (Figure 1).

### Haemodynamics

**Figure 2:**
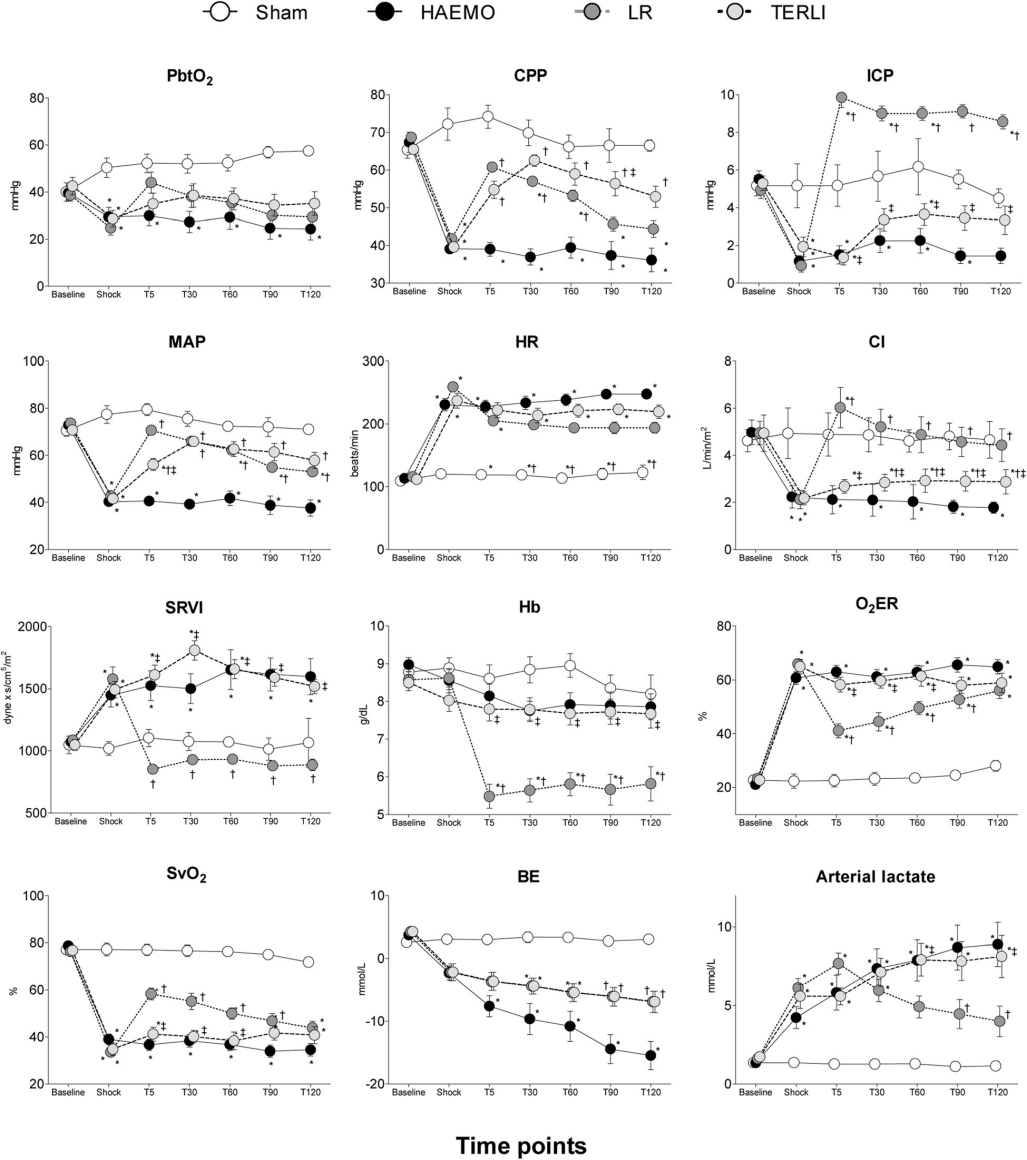
**Effects of terlipressin and lactated Ringer’s solution in a porcine model of haemorrhagic shock.** LR, Rats treated with lactated Ringer’s solution after haemorrhagic shock; TERLI, Rats treated with terlipressin after haemorrhagic shock; HAEMO, Non-treated rats; Sham, Rats not subjected to bleeding; PbtO_2_, Brain tissue oxygen tension; CPP, Cerebral perfusion pressure; ICP, Intracranial pressure; MAP, Mean arterial pressure; HR, Heart rate; CI, Cardiac index; SRVI, Systemic resistance vascular index; Hb, Arterial haemoglobin level; O_2_ER, Oxygen extraction ratio; SvO_2_, Mixed venous oxygen saturation; BE, Base excess. Data are expressed as mean ± standard error. *P <0.05 versus sham group. ^†^ *P* <0.05 versus HAEMO group. ^‡^ *P* <0.05 versus LR group.

HR was significantly increased in the HAEMO, LR and TERLI groups from the time of shock to T120 compared with the sham group (*P* <0.001). From T30 to T120, HR was significantly lower in the LR group than in the HAEMO group (*P* <0.05) (Figure 2). MAP was significantly decreased at shock in all study groups compared with the sham group (*P* <0.001). In the HAEMO group, the MAP was significantly decreased from T5 to T120 compared with the other groups (*P* <0.001). Compared with sham animals, MAP was significantly decreased at T60, T90 and T120 in the LR group (*P* <0.001) and at T5 in the TERLI group (*P* <0.001). No significant differences in this variable were observed from T30 to T120 between the LR and TERLI groups (Figure 2). The CI was significantly decreased from T5 to T120 in the HAEMO and TERLI groups compared with the sham group (*P* <0.01). At the corresponding time points, the CI was significantly higher in the TERLI group than in the HAEMO group (*P* <0.05). In the LR group, CI was higher than in the HAEMO and TERLI groups (*P* <0.05) (Figure 2).

RAP was significantly decreased (*P* <0.001) and SVRI and PVRI were significantly increased (*P* <0.01) from shock to T120 in the HAEMO and TERLI groups compared with the sham group (*P* <0.01), whereas no significant differences were observed in these variables from T5 to T120 between the LR and sham groups. MPAP was significantly decreased in all groups from T5 to T120 compared with the sham group (*P* <0.05) (Figure 2 and Table 1). In all study groups, PAOP was significantly decreased at shock compared with the sham group. In the LR group, PAOP was significantly increased at T5 and T120 compared with the HAEMO group at these corresponding time points (Table 1). LVSWI, RVSWI and SVI were significantly decreased in the LR and TERLI groups compared with the sham group (*P* <0.01) and significantly increased in the HAEMO group, compared with the sham group (*P* <0.01) (Table 1).

**Table 1:** **Effects of haemorrhagic shock on haemodynamics at baseline, shock and at 5, 30, 60, 90 and 120 posttreatment**

		**Baseline**	**Shock**	**After treatment (min)**				
				**5**	**30**	**60**	**90**	**120**
RAP (mmHg)								
	Sham	8 ± 1	9 ± 2	8 ± 1	8 ± 1	9 ± 1	7 ± 1	6 ± 0
	HAEMO	7 ± 2	2 ± 1*	2 ± 1*	2 ± 1*	2 ± 1*	2 ± 1*	2 ± 1*
	LR	7 ± 1	2 ± 1*	8 ± 2^†^	7 ± 2^†^	6 ± 2*^†^	5 ± 2^†^	5 ± 2^†^
	TERLI	7 ± 2	2 ± 1*	2 ± 1*^‡^	3 ± 1*^‡^	3 ± 1*^‡^	2 ± 1*^‡^	2 ± 1*^‡^
MPAP (mmHg)								
	Sham	19 ± 1	20 ± 1	20 ± 1	20 ± 1	20 ± 1	18 ± 1	18 ± 3
	HAEMO	20 ± 3	16 ± 2*	17 ± 3*	17 ± 3*	17 ± 4*	16 ± 2*	16 ± 2*
	LR	19 ± 2	17 ± 2*	22 ± 5*^†^	20 ± 4^†^	19 ± 3^†^	19 ± 2^†^	19 ± 2^†^
	TERLI	20 ± 1	16 ± 2*	18 ± 3^†‡^	18 ± 3^†^	18 ± 2^†^	19 ± 2^†^	19 ± 2^†^
PAOP (mmHg)								
	Sham	12 ± 1	11 ± 2	11 ± 2	11 ± 1	11 ± 2	9 ± 1	11 ± 2
	HAEMO	11 ± 1	8 ± 2*	8 ± 2*	8 ± 1*	8 ± 1*	7 ± 1*	7 ± 1*
	LR	11 ± 1	9 ± 2*	11 ± 2^†^	10 ± 2	9 ± 2	9 ± 2	10 ± 2^†^
	TERLI	11 ± 1	8 ± 1*	8 ± 2	9 ± 1	9 ± 1	9 ± 1	9 ± 1
LVSWI (g-m/m^2^/beat)								
	Sham	30 ± 7	35 ± 13	35 ± 10	35 ± 11	33 ± 6	32 ± 6	29 ± 5
	HAEMO	34 ± 6	4 ± 1*	3 ± 1*	3 ± 1*	3 ± 1*	3 ± 1*	3 ± 1*
	LR	35 ± 4	4 ± 1*	19 ± 3*^†^	15 ± 4*^†^	14 ± 4*^†^	13 ± 4*^†^	12 ± 4*^†^
	TERLI	37 ± 7	4 ± 1*	8 ± 2*^†^	10 ± 3*^†^	10 ± 3*^†^	9 ± 3*^†^	8 ± 4*^†^
RVSWI (g × m/m^2^/beat)								
	Sham	6 ± 0	7 ± 1	6 ± 1	6 ± 1	5 ± 1	7 ±	6 ± 0
	HAEMO	7 ± 1	2 ± 0*	2 ± 0*	1 ± 0*	1 ± 0*	1 ± 0*	1 ± 0*
	LR	7 ± 1	2 ± 0*	6 ± 2*^†^	5 ± 2*^†^	5 ± 2*^†^	5 ± 1*^†^	5 ± 1*^†^
	TERLI	8 ± 1	2 ± 1*	3 ± 1*†	3 ± 0*^†^	3 ± 1*^†^	3 ± 0*^†^	3 ± 1*^†^
SVI (ml/beat/m^2^)								
	Sham	44 ± 11	40 ± 11	38 ± 11	39 ± 13	41 ± 8	41 ± 9	38 ± 11
	HAEMO	42 ± 6	8 ± 2*	8 ± 2*	7 ± 1*	7 ± 1*	7 ± 1*	7 ± 1*
	LR	43 ± 4	8 ± 1*	28 ± 4*^†^	25 ± 5*^†^	24 ± 6*^†^	24 ± 6*^†^	23 ± 6*^†^
	TERLI	47 ± 7	9 ± 1*	12 ± 3*	13 ± 2*^†^	13 ± 2*^†^	13 ± 3*^†^	13 ± 3*^†^

LR, Rats treated with lactated Ringer’s solution after haemorrhagic shock; TERLI, Rats treated with terlipressin after haemorrhagic shock; HAEMO, Non-treated rats; Sham, Rats not subjected to bleeding; RAP, Right atrial pressure; MPAP, Mean pulmonary artery pressure; PAOP, Pulmonary artery occlusion pressure; LVSWI, Left ventricular stroke work index; RVSWI, Right ventricular stroke work index; SVI, Stroke volume index. *P <0.05 versus sham group. ^†^ *P* <0.05 versus HAEMO group. ^‡^ *P* <0.05 versus LR group.

### Blood gases, oxygenation and electrolytes

pH, HCO_3_^−^, BE, SvO_2_ and DO_2_I were significantly decreased, and O_2_ER, arterial lactate and K^+^ were significantly increased, from shock to T120 in the HAEMO, LR and TERLI groups compared with the sham group (*P* <0.05) (Table 2). In the LR and TERLI groups, DO_2_I was significantly increased from T5 to T120 and from T30 to T120, respectively, compared with the HAEMO group (*P* <0.05). In the LR and TERLI groups, the VO_2_I was significantly increased from T5 to T120 compared with the sham group (*P* <0.05) and significantly increased from T30 to T120 compared with the HAEMO group (*P* <0.05). The levels of Hb and Na^+^ were significantly lower from T5 to T120 in the LR group compared with the sham group (*P* <0.05). The ratio of arterial oxygen partial pressure to fractional inspired oxygen and the level of PaCO_2_ did not change significantly in any group during the study (Figure 2 and Table 2).

**Table 2:** **Effects of haemorrhagic shock on blood gases, oxygenation and electrolytes at baseline, shock and at 5, 30, 60, 90 and 120 minutes posttreatment**

		**Baseline**	**Shock**	**After treatment (min)**				
				**5**	**30**	**60**	**90**	**120**
PaCO_2_ (mmHg)								
	Sham	47 ± 0	43 ± 3	41 ± 2	42 ± 1	42 ± 0	40 ± 0	41 ± 1
	HAEMO	42 ± 3	39 ± 4	35.6 ± 7*	43 ± 8	38 ± 6	38 ± 8	38 ± 8
	LR	42 ± 4	38 ± 4	42.3 ± 3	41 ± 3	40 ± 2	39 ± 2	39 ± 2
	TERLI	43 ± 2	42 ± 4	42.9 ± 5^†^	47 ± 6^†^	48 ± 7^†^	45 ± 4^†^	44 ± 5
DO_2_I (ml/min/m^2^)								
	Sham	540 ± 2	571 ± 107	501 ± 105	522 ± 108	553 ± 64	543 ± 95	518 ± 131
	HAEMO	597 ± 81	243 ± 52*	212 ± 64*	190 ± 37*	192 ± 38*	194 ± 46*	190 ± 37*
	LR	580 ± 61	237 ± 73*	400 ± 49*^†^	375 ± 62*^†^	361 ± 66*†	344 ± 64*^†^	339 ± 59*^†^
	TERLI	581 ± 96	238 ± 27*	288 ± 19*	307 ± 43*^†^	313 ± 51*†	302 ± 55*^†^	299 ± 73*^†^
VO_2_I (ml/min/m^2^)								
	Sham	121 ± 3	140 ± 17	126 ± 28	133 ± 13	139 ± 9	133 ± 21	143 ± 24
	HAEMO	126 ± 27	154 ± 36	139 ± 40	119 ± 20	123 ± 39	126 ± 27	122 ± 21
	LR	129 ± 33	152 ± 38	173 ± 37*	176 ± 24*^†^	182 ± 30*†	178 ± 24*^†^	187 ± 33*^†^
	TERLI	123 ± 19	152 ± 21	161 ± 8*	174 ± 22^*†^	176 ± 23*†	172 ± 23*^†^	170 ± 24*^†^
K^+^ (mmol/L)								
	Sham	4.4 ± 0.3	4.3 ± 0.0	4.0 ± 0.1	4.3 ± 0.1	4.4 ± 0.1	4.4 ± 0.5	4.5 ± 0.3
	HAEMO	4.3 ± 0.4	5.6 ± 0.9*	5.2 ± 0.9	5.7 ± 0.9*	5.8 ± 0.8*	6.0 ± 0.6*	6.2 ± 0.5*
	LR	3.9 ± 0.2	5.8 ± 1.5*	4.0 ± 0.3^†^	4.4 ± 0.4^†^	4.9 ± 0.6*†	5.2 ± 0.7*	5.1 ± 1.1*
	TERLI	4.2 ± 0.2	5.3 ± 0.5*	5.4 ± 0.5*	5.0 ± 0.5	5.2 ± 0.5*	5.3 ± 0.6*	5.5 ± 0.7*
Na^+^ (mmol/L)								
	Sham	140 ± 1	140 ± 0	142 ± 1	140 ± 3	141 ± 1	139 ± 3	139 ± 1
	HAEMO	139 ± 3	137 ± 2	137 ± 3	137 ± 2	137 ± 2	137 ± 2	136 ± 2
	LR	141 ± 2	138 ± 4	137 ± 3	137 ± 3*	136 ± 2*	135 ± 3*	135 ± 2*
	TERLI	140 ± 3	139 ± 3	137 ± 2	138 ± 3	138 ± 3	139 ± 3	139 ± 5

LR, Rats treated with lactated Ringer’s solution after haemorrhagic shock; TERLI, Rats treated with terlipressin after haemorrhagic shock; HAEMO, Non-treated rats; Sham, Rats not subjected to bleeding; PaCO_2_, Partial pressure of dioxide carbon in the arterial blood; DO_2_I, Oxygen delivery index; VO_2_I, Oxygen consumption index; K^+^, Potassium ion levels; Na^+^, Sodium ion levels. *P <0.05 versus sham group. ^†^ *P* <0.05 versus HAEMO group.

### Neuromonitoring

CPP, ICP and PbtO_2_ were significantly decreased from shock to T60 in the HAEMO group compared with the sham animals (*P* <0.05). Both treatments with LR and TERLI were followed by a significant increase in CPP compared with the HAEMO group (*P* <0.01), with CPP recovering to values not significantly different from those of the sham group. The LR group had the largest increase in ICP, which was observed from T5 to T120 (*P* <0.05 versus sham; *P* <0.001 versus HAEMO group; *P* <0.001 versus TERLI group). The TERLI group had no significant differences in ICP from T30 to T120 compared with the sham group. Treatments with LR and TERLI recovered PbtO_2_ to values similar to those in the sham group (Figure 2).

### Aquaporin-4 and Na^+^-K^+^-2Cl^−^ co-transporter

**Figure 3:**
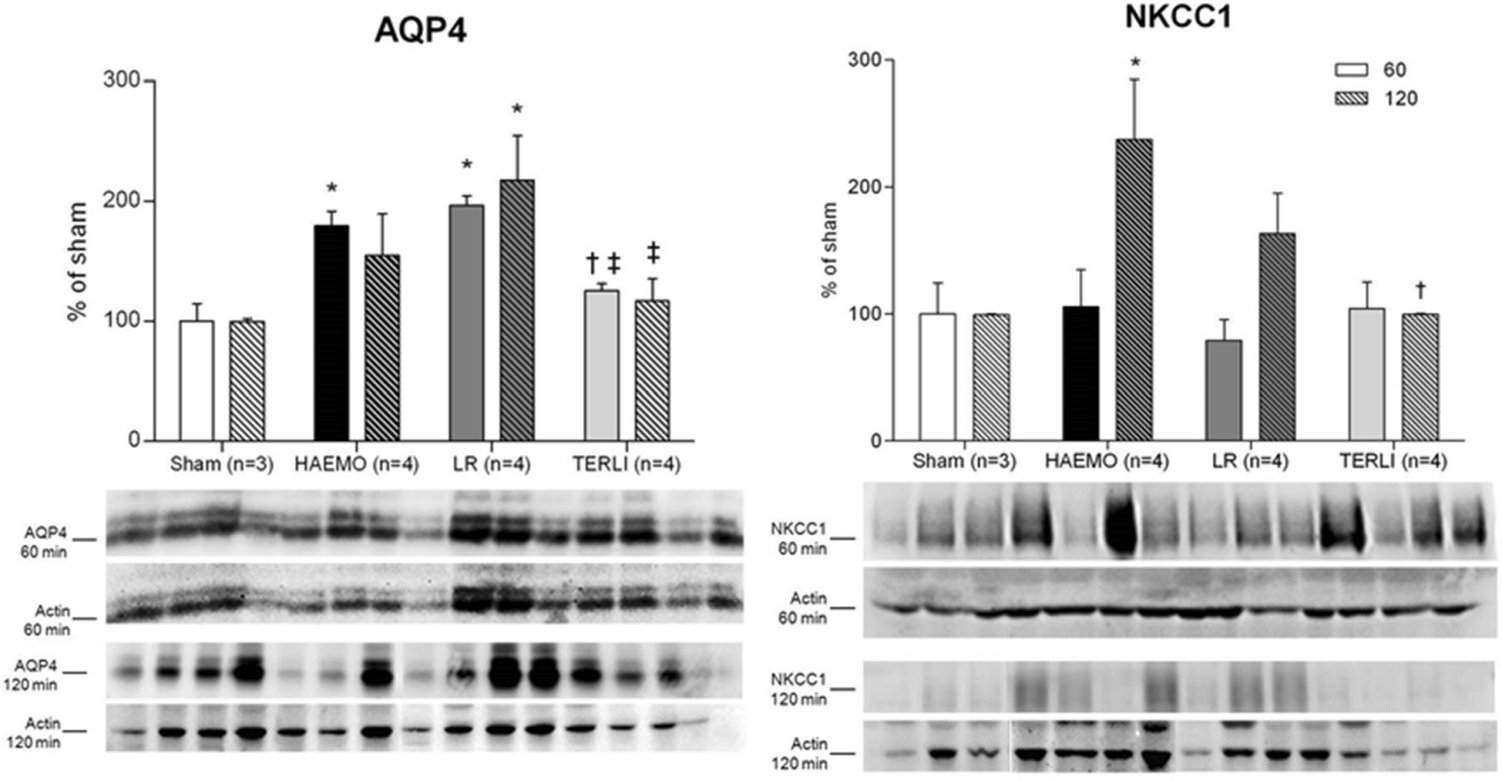
**Semiquantitative immunoblotting of membrane fractions prepared from cerebral samples.** A densitometric analysis for the expression of aquaporin-4 (AQP4) and Na^+^-K^+^-2Cl^−^ co-transporter (NKCC1) in samples from the sham, HAEMO, LR and TERLI groups is shown. Immunoblots reacted with anti-AQP4 revealed a 34 kDa band, and those reacted with anti-NKCC1 revealed a 135 to 170 kDa band. Data are expressed as mean ± standard error. *P <0.05 versus sham group. ^†^ *P* <0.05 versus HAEMO group. ^‡^ *P* <0.05 versus LR group.

At 60 minutes after shock, semiquantitative immunoblot analysis revealed a significant increase in the expression of AQP4 in the HAEMO group (179 ± 12% of sham, *P* =0.0086), which was not reversed by treatment with LR (196 ± 8% of sham, *P* =0.0047), but was fully restored by TERLI (125 ± 6% of sham). In the TERLI group, the expression of AQP4 was significantly higher at 60 minutes compared with the HAEMO and LR groups (*P* =0.0071). At 120 minutes, a significant upregulation of AQP4 was observed only in the LR group (217 ± 37% of sham, *P* =0.0084), which was significantly higher than in TERLI group (117 ± 19% of sham, *P* =0.0169) (Figure 3). No significant increase in the expression of NKCC1 was observed in any group at 60 minutes after shock, but NKCC1 expression was significantly increased at 120 minutes in the HAEMO group (237 ± 47% of sham, *P* =0.0234), which was fully restored by treatment with terlipressin (100 ± 1% of sham, *P* =0.0270) (Figure 3).

### Manganese superoxide dismutase and thiobarbituric acid reactive substances

**Figure 4:**
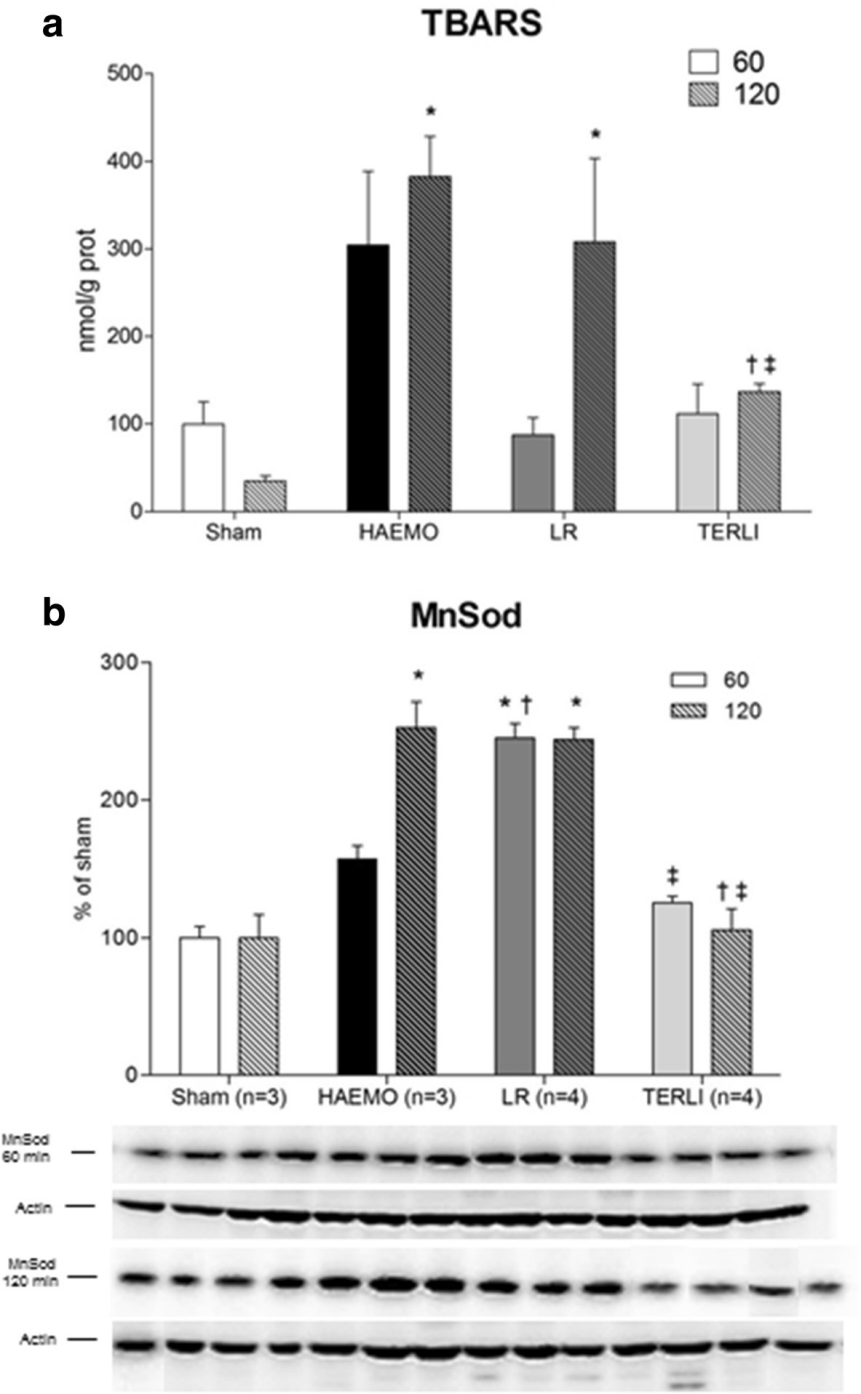
**Changes in the level of thiobarbituric acid reactive substances and expression of manganese superoxide dismutase from cerebral samples from the sham, HAEMO, LR and TERLI groups. (a)** Each bar represents the group mean ± standard error. **(b)** Immunoblots reacted with antibody against manganese superoxide dismutase revealing a 24 kDa band. *P <0.05 versus sham group. ^†^ *P* <0.05 versus HAEMO group. ^‡^ *P* <0.05 versus LR group. HAEMO, No-treatment group; LR, Lactated Ringer’s solution group; TERLI, Terlipressin group.

The levels of TBARS were not significantly different from the sham group in any study group at 60 minutes after shock. However, at 120 minutes after shock, these levels were clearly higher in the HAEMO group (0.38 ± 0.05 nmol/mg of protein; *P* =0.0013) and LR group (0.31 ± 0.10 nmol/mg of protein; *P* =0.0167), but not in the TERLI group (0.14 ± 0.01 nmol/mg of protein) compared with the sham group (0.03 ± 0.01 nmol/mg of protein). At 120 minutes after shock, the levels of TBARS in the TERLI group were significantly lower than in the HAEMO group (*P* <0.0001) and the LR group (*P* =0.0394) (Figure 4). Animals treated with LR had the highest expression of MnSOD at 60 minutes after shock (245 ± 11% of sham, *P* <0.0001), whereas no significant changes in the expression of MnSOD were observed in the other groups at the corresponding time point (HAEMO group: 157 ± 10% of sham; TERLI group: 125 ± 5% of sham). At 120 minutes after shock, the expression of MnSOD was significantly increased in the HAEMO group (237 ± 14% of sham, *P* =0.0081), which was not reversed by LR (244 ± 9% of sham, *P* =0.0009), but it was fully restored by TERLI (105 ± 16% of sham) (Figure 4).

### Bax and Bcl-x

**Figure 5:**
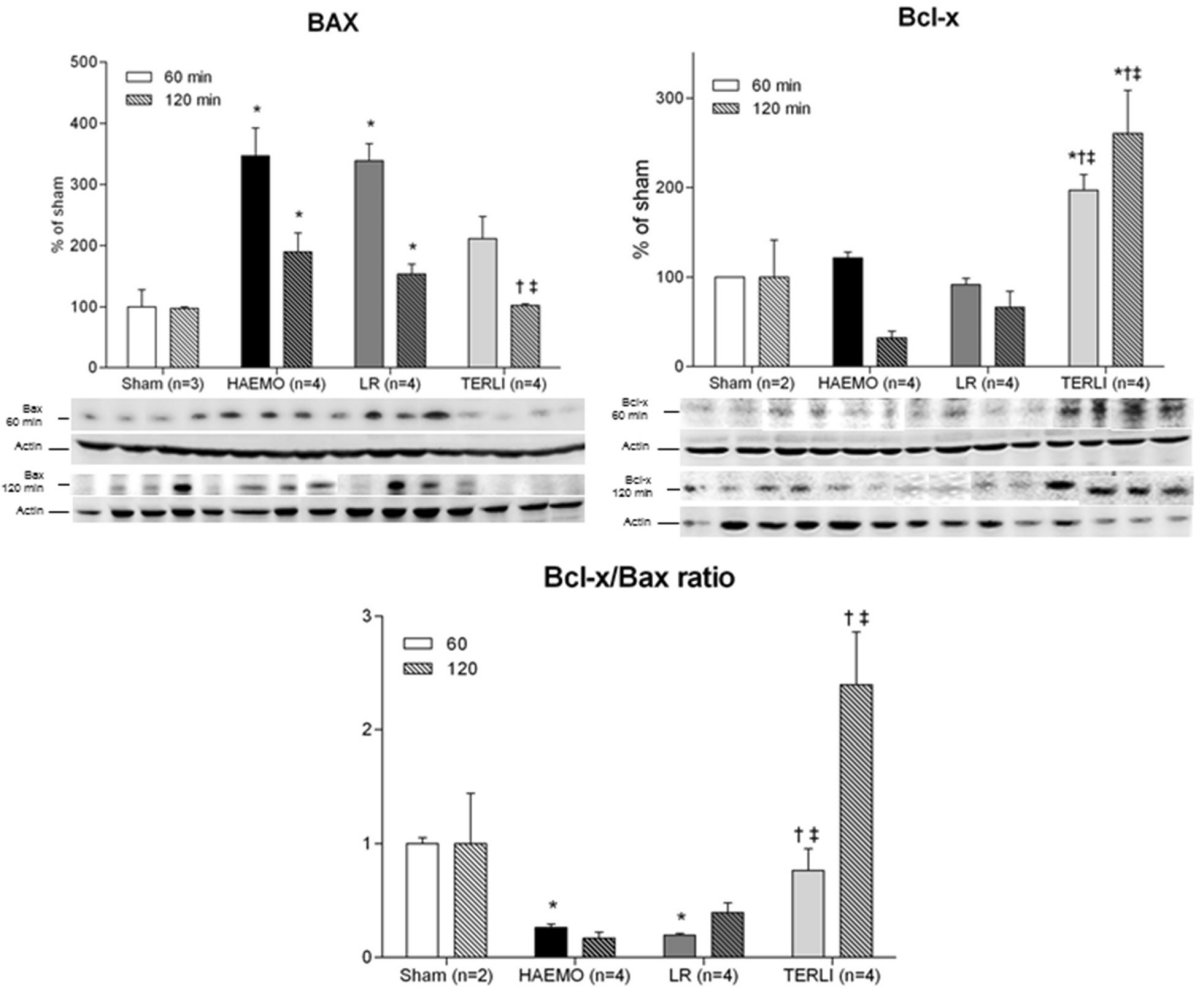
**Semiquantitative immunoblotting of membrane fractions prepared from cerebral samples.** A densitometric analysis for the expression of Bax and Bcl-x in samples from the sham, HAEMO, LR and TERLI groups is shown. Immunoblots reacted with pro- and antiapoptotic Bax and Bcl-x proteins revealed 23 kDa and 26 kDa bands, respectively. Data are expressed as mean ± standard error. *P <0.05 versus sham group. ^†^ *P* <0.05 versus HAEMO group. ^‡^ *P* <0.05 versus LR group. HAEMO, No-treatment group; LR, Lactated Ringer’s solution group; TERLI, Terlipressin group.

The antiapoptotic Bcl-x protein was significantly upregulated at 60 and 120 minutes after shock in the TERLI group (60 minutes: 197 ± 17% of sham, *P* =0.0038; 120 minutes: 261 ± 48% of sham, *P* =0.0033), but not in the HAEMO group (60 minutes: 122 ± 6% of sham; 120 minutes: 32 ± 8% of sham) or LR group (60 minutes: 92 ± 7% of sham; 120 minutes: 67 ± 18% of sham) (Figure 5). The proapoptotic Bax protein was markedly upregulated at 60 and 120 minutes in the HAEMO group (60 minutes: 347 ± 46% of sham, *P* =0.0088; 120 minutes: 190 ± 31% of sham, *P* =0.0154) and LR group (60 minutes: 339 ± 28% of sham, *P* =0.0021; 120 minutes: 154 ± 16% of sham, *P* =0.0129), significantly higher than the TERLI group (Figure 5). The Bcl-x/Bax ratio was significantly decreased at 60 minutes after shock in the HAEMO group (0.26 ± 0.03, *P* =0.0002) and LR group (0.20 ± 0.02, *P* <0.0001) compared with the sham group (1.00 ± 0.05). At the corresponding time, animals treated with TERLI had a Bcl-x/Bax ratio (0.77 ± 0.19) significantly higher than that of the HAEMO group (*P* =0.0393) and LR group (*P* =0.0235). At 120 minutes after shock, the Bcl-x/Bax ratio was significantly higher in the TERLI group (2.40 ± 0.46) than in the HAEMO group (0.17 ± 0.05, p =0.0098) and LR group (0.40 ± 0.08, *P* =0.0054), but not with that in the sham group (1.00 ± 0.44) (Figure 5).

## Discussion

Haemorrhagic shock can result in global cerebral hypoxia caused by hypovolaemia and hypotension, and the haemodynamic resuscitation must restore CPP in order to prevent ischaemic injury within the brain [6-8,26]. The results of the present study indicate that early treatment with terlipressin can recover CPP after haemorrhagic shock and that the underlying mechanisms include regulation of water and Na^+^ channels, inhibition of oxidative stress and decrease of apoptotic signalling within the brain. Survival times were similar at 120 minutes after haemorrhagic shock among groups, with the exception of the HAEMO group. However, whereas therapy with TERLI provided superior outcomes than LR with regard to measures of cerebral damage, it provided inferior outcomes with regard to systemic or peripheral measures of haemodynamics and tissue perfusion.

As expected, CPP was not preserved at a blood pressure below the cerebral autoregulation threshold [27], which was followed by reduction in PbtO_2_ [8,28,29]. In the non-treated animals, these derangements were associated with upregulation of AQP4 and NKCC1. These proteins play an important role in the formation of cellular oedema in the brain by regulating water and Na^+^ transport through the sealing junctions of the blood–brain barrier in response to cerebral ischaemia [10,30-32]. AQP4 acts by increasing water transport mainly in the pericapillary foot process of astrocytes [9], and NKCC1 acts by increasing secretion of Na^+^, Cl^−^ and water through an intact blood–brain barrier into the brain [10]. Therefore, it was suggested that cerebral hypoperfusion was followed by ischaemic lesions in the HAEMO group. Cerebral hypoperfusion might have contributed to an accumulation of lipid peroxidation products, as reflected by the increased levels of TBARS, which might induce a compensatory increase in the expression of MnSOD [12,33,34]. Oxygen free radicals exert their pathophysiologic effects by directly attacking lipids and proteins in the biologic membranes, which can cause cellular dysfunction and induce apoptotic cell death [35]. Indeed, in the present study, the exposure of the brain to a high level of oxidative stress following haemorrhagic shock was associated with a marked shift in the Bcl-x/Bax ratio, indicating a loss of antiapoptotic ability [36]. Bcl-x and Bax are proteins that play an important role in determining the relative sensitivity of neuronal subpopulations to ischaemia. Accordingly, previous studies showed that haemorrhagic shock can induce a significant oxidative stress in the brain [12,37] and that cerebral ischaemia can decrease the Bcl-x/Bax ratio [38]. Furthermore, other markers of cerebral cellular damage have also been described following haemorrhagic shock in other studies, such as an increased level of glycerol in brain tissue [26] and increased plasma levels of S100B [7], findings which support the presently reported results.

The direct vasoconstrictive effect of terlipressin, reflected by increased MAP and SRVI, prevented an improvement in CI but probably allowed for the redistribution of blood flow towards the cerebral circulation, leading to the restoration of CPP and PbtO_2_. Some effects cannot be differentiated from the common properties of any vasopressor, but it has been suggested that terlipressin can induce a selective vasoconstrictor effect according to the distribution of V_1_ vasopressin receptors. This hypothesis is supported by previous studies in which researchers used norepinephrine in models of haemorrhagic shock, which did not improve CPP or oxygenation [39,40]. Furthermore, another study showed that vasopressin, the natural terlipressin analogue, resulted in a significantly higher increase of CPP compared with norepinephrine [41]. As with the TERLI group, an increased SRVI was also found in non-treated animals; however, this was not accompanied by an increase in MAP in the HAEMO group. The increase in MAP could have restored the cerebral autoregulation in animals treated with terlipressin, which might explain the recovery in CPP and PbtO_2_. Another explanation for the recovery in CPP is the higher PaCO_2_ compared with the HAEMO group. Because PaCO_2_ has a linear positive correlation with cerebral blood flow [42], it could account for more cerebral vasodilation and hence better perfusion compared with non-treated animals. However, it was unclear whether terlipressin also acted directly via V_1_ vasopressin receptors within the brain [43], which might also explain the recovery in ICP and, in turn, CPP. Moreover, the expression levels of both AQP4 and NKCC1 were also restored in animals treated with terlipressin, which also supports the finding that cerebral perfusion was recovered. Despite the increase in SRVI, the fact that lactate, O_2_ER and SvO_2_ were not significantly different between groups suggests that terlipressin did not impair peripheral perfusion compared with the HAEMO group. In fact, BE was less negative in the TERLI group. However, compared with the treatment with LR, terlipressin resulted in inferior outcomes with regard to measures of haemodynamics such as CI, RAP, SVRI and PVRI, which might explain the death of one animal in the TERLI group.

As with the present data, an improvement in CPP has also been described in patients with persistent arterial hypotension and acute liver failure [17], traumatic brain injury [23] and septic shock [22] who were successfully treated with terlipressin. In the present study, systemic hypoperfusion could have accounted for unreleased, and thus undetected, products of oxidative stress within the brain, which could have caused cell damage. However, if that was the case, then PbtO_2_ would not have recovered, unless mitochondria were incapable of using oxygen, allowing an increased availability of oxygen within the tissue. As mitochondrial function was not assessed in the present study, this explanation remains speculative. Also, oxidative damage would probably be associated with inflammation, but this was not supported by a previous study in which researchers found an improved inflammatory cytokine profile in rats treated with terlipressin compared with LR [14]. Nonetheless, the outcome was associated with an increase in the Bcl-x/Bax ratio, suggesting that if any cerebral oxidative injury or ischaemia were present, it probably was not sufficiently severe to trigger antiapoptotic signalling [36,38] within the brain in terlipressin-treated animals.

Treatment with LR, however, was followed by a discrepancy between the increments in PbtO_2_ that was not accompanied by recovery of CPP. This discrepancy can be attributed to the systemic third-spacing of crystalloids, which would also be consistent with the decline in MAP over the course of 120 minutes [14]. This explanation is supported by the overexpression of AQP4 in the LR group, which might indicate a compensatory mechanism to eliminate excess water within the brain water [44]. An increase in brain water content can also explain the increase in ICP, which, in turn, is detrimental to the restoration of CPP. This finding is also in line with the decreased blood Na^+^ levels in the group treated with LR compared with the sham group. Another hypothesis is that a decrease in blood viscosity after intravascular volume expansion, despite some differences in ICP, could increase cerebral blood flow [45] and thus explain the similar PbtO_2_ values between LR and TERLI groups. The LR group had the largest increase in ICP, but whether it induced overexpression of the brain tissue markers of water balance, oxidative stress and apoptosis is unknown. The fact is that regardless of the mechanism(s) underlying the failure of full recovery of CPP in the LR group, it remains the case that the markers of oxidative stress, TBARS and MnSOD, were overexpressed in animals treated with LR. The fluid infusion could have carried the overproduction of reactive oxygen species (ROS) throughout the tissue initiating a postischaemic reperfusion injury [36,46,47]. ROS regulate mechanisms via inflammatory pathways that ultimately can decrease vascular resistance [35], allowing for an increase in brain volume that could be partly responsible for unrecovered CPP in the LR group. In fact, researchers in a previous study found an increased proinflammatory cytokine profile and severe hypotension (40 mmHg) in rats treated with LR after haemorrhagic shock [14]. This oxidative stress could have reduced the antiapoptotic trend of the Bcl-x/Bax ratio in the brain during postischaemic reperfusion [36,38]. These alterations in the expression of Bcl-x and Bax may indicate that mitochondria were dysfunctional, as these proteins are part of the intrinsic mitochondria-related apoptotic pathway. Therefore, similar to the considerations previously described for the terlipressin-treated animals, one hypothesis is that an increased PbtO_2_ could have been caused by an increased availability of oxygen because dysfunctional mitochondria are not capable of using the oxygen available in the tissue.

Some limitations of this study should be noted. First is the short observation time, which we used because the experiment was designed to determine the early cerebral effects observed during prehospital care, rather than to determine long-term functional neurologic outcome or correlation to brain histopathology. Second, PbtO_2_ was measured locally with a probe placed in the cerebral cortex, and therefore global ischaemia caused by a heterogeneous distribution of PbtO_2_ may have been underestimated with regard to the brain region analysed. Third, cerebral blood flow was not measured, because the purpose of the present study was to evaluate changes in CPP and oxygenation. Also, the anaesthetics used may be cerebroprotective and may have different effects in ICP and MAP, but these were minimised by having a sham group and another group not treated after haemorrhagic shock, which were both subjected to the same anaesthetic protocol as the treated groups. In addition, some varieties caused by different vasopressin receptors in pigs (lysine vasopressin) and humans (arginine vasopressin) might have resulted in a different haemodynamic response to terlipressin. However, these differences do not interfere with the results of our study, because the study was aimed at investigating differences between groups and changes over time rather than presenting absolute values. Finally, we used only terlipressin as a vasopressor, and therefore we are unable to report whether different vasopressors would have yielded other results.

## Conclusions

Early treatment with terlipressin was effective at restoring CPP and preventing dysregulation of water balance, and oxidative and apoptotic markers within the brain, following haemorrhagic shock in our model. These results indicate that the role of this pressor agent on brain perfusion in haemorrhagic shock requires further investigation.

## Key messages

- Early treatment with terlipressin recovered cerebral perfusion pressure and brain tissue oxygen tension after haemorrhagic shock in pigs.
- Terlipressin was effective for normalising cerebral markers of water balance, oxidative damage and apoptosis after haemorrhagic shock.
- These cerebral improvements were observed for at least 2 hours after haemorrhagic shock in animals treated with terlipressin.

## Abbreviations

ANOVA: Analysis of variance
AQP4: Aquaporin-4
BE: Base excess
CI: Cardiac index
CO: Cardiac output
CPP: Cerebral perfusion pressure
DO_2_I: Systemic oxygen delivery index
ECL: Enhanced chemiluminescence
HAEMO: No-treatment group
Hb: Haemoglobin
HR: Heart rate
HS: Haemorrhagic shock
ICP: Intracranial pressure
IgG: Immunoglobulin G
LR: Lactated Ringer’s solution group
LVSWI: Left ventricular stroke work index
MAP: Mean arterial blood pressure
MnSOD: Manganese superoxide dismutase
MPAP: Mean pulmonary artery pressure
NKCC1: Na^+^-K^+^-2Cl^−^ co-transporter
O_2_ER: Oxygen extraction ratio
PaCO_2_: Partial pressure of carbon dioxide in the arterial blood
PAOP: Pulmonary artery occlusion pressure
PbtO_2_: Brain tissue oxygen tension
PVRI: Pulmonary vascular resistance index
RAP: Right atrial pressure
ROS: Reactive oxygen species
RVSWI: Right ventricular stroke work index
SVI: Stroke volume index
SvO_2_: Mixed-venous oxygen saturation
SVRI: Systemic vascular resistance index
TBARS: Thiobarbituric acid reactive substance
TERLI: Terlipressin group
VO_2_I: Systemic oxygen consumption
